# Patients Living With Arterial Hypertension in Mexico: First Insights of The Mexican Registry of Arterial Hypertension (RIHTA Study)

**DOI:** 10.1093/ajh/hpae024

**Published:** 2024-03-11

**Authors:** Silvia Palomo-Piñón, Neftali Eduardo Antonio-Villa, Luis Rey García-Cortés, Moises Moreno-Noguez, Luis Alcocer, Humberto Álvarez-López, Ernesto G Cardona-Muñoz, Adolfo Chávez-Mendoza, Enrique Díaz-Díaz, José Manuel Enciso-Muñoz, Héctor Galván-Oseguera, Martín Rosas-Peralta, Mexican Group of Experts on Arterial Hypertension, Silvia Palomo-Piñón, Neftali Eduardo Antonio-Villa, Luis Rey García-Cortés, Luis Alcocer, Humberto Álvarez López, Ernesto G Cardona-Muñoz, Adolfo Chávez-Mendoza, Enrique Díaz-Díaz, Héctor Galván-Oseguera, Martin Rosas-Peralta, Moises Moreno-Noguez, Maria de los Ángeles Dichi Romero, Pedro Luis Vargas Gutiérrez, Maria Eugenia Figueroa Suárez, Rubén Rios Morales, Francisco Vargas Hernández, Irma Fabiola García Padilla, Alfonso Zempoalteca Morales, Imer Guillermo Herrera Olvera, Gloria Mendoza López, Ana Laura Guerrero Morales, María Elisa López Delgado, Ana Lilia Gonzales Ramírez, Jairo Enoc Cruz Toledo, Olivia Reyes Jiménez, Diana Amaya Mora, Isaac Pérez Zamora, Flor Araceli Nava Ayala, Tabata Gabriela Anguiano Velázquez, Oscar Jiménez Jalpa, Ma Adriana Cruz Arce, Vidal José González Coronado

**Affiliations:** Grupo de Expertos en Hipertensión Arterial México (GREHTA), Ciudad de México, México; Grupo Colaborativo en Hipertensión Arterial (GCHTA), Ciudad de México, México; Departamento de Endocrinología, Instituto Nacional de Cardiología Ignacio Chávez, Ciudad de México, México; Coordinación de Planeación y Enlace Institucional, Jefatura de Servicios de Prestaciones Médicas, Órgano de Operación Administrativa Desconcentrada Regional Estado de México Oriente, Instituto Mexicano del Seguro Social, Estado de México, Oriente, México; Grupo de Expertos en Hipertensión Arterial México (GREHTA), Ciudad de México, México; Coordinación Clínica de Educación e Investigación en Salud, Unidad de Medicina Familiar No. 55 Zumpango, Órgano de Operación Administrativa Desconcentrada Regional Estado de México Oriente, Estado de México, México; Grupo de Expertos en Hipertensión Arterial México (GREHTA), Ciudad de México, México; Hospital Ángeles del Pedregal, Ciudad de México, México; Grupo de Expertos en Hipertensión Arterial México (GREHTA), Ciudad de México, México; Hospital Puerta de Hierro Andares, Zapopan, Jalisco, México; Grupo de Expertos en Hipertensión Arterial México (GREHTA), Ciudad de México, México; Centro Universitario de Ciencias de la Salud, Universidad de Guadalajara, Guadalajara, Jalisco, México; Grupo de Expertos en Hipertensión Arterial México (GREHTA), Ciudad de México, México; Unidad Médica de Alta Especialidad de Cardiología, Centro Médico Nacional SXXI, Instituto Mexicano del Seguro Social, Ciudad de México, México; Grupo de Expertos en Hipertensión Arterial México (GREHTA), Ciudad de México, México; Unidad Médica de Alta Especialidad de Cardiología, Centro Médico Nacional SXXI, Instituto Mexicano del Seguro Social, Ciudad de México, México; Grupo de Expertos en Hipertensión Arterial México (GREHTA), Ciudad de México, México; Hospital General Zacatecas "Luz González Cosio", Instituto de Seguridad y Servicios Sociales de los Trabajadores del Estado, Zacatecas, Zacatecas, México; Asociación Nacional de Cardiólogos de México, Ciudad de México, México; Grupo de Expertos en Hipertensión Arterial México (GREHTA), Ciudad de México, México; Unidad Médica de Alta Especialidad de Cardiología, Centro Médico Nacional SXXI, Instituto Mexicano del Seguro Social, Ciudad de México, México; Grupo de Expertos en Hipertensión Arterial México (GREHTA), Ciudad de México, México; Titular Academia Nacional de Medicina, Ciudad de México, México

**Keywords:** arterial hypertension, blood pressure, epidemiology, hypertension, hypertension management, Mexico

## Abstract

**BACKGROUND:**

Arterial hypertension is a significant cause of morbidity and mortality in Mexico. However, there is limited evidence to understand blood pressure management and cardiometabolic profiles. Here, we aim to assess the prevalence of controlled and uncontrolled blood pressure, as well as the prevalence of cardiometabolic risk factors among patients from the Mexican Registry of Arterial Hypertension (RIHTA).

**METHODS:**

We conducted a cross-sectional analysis of participants living with arterial hypertension registered on RIHTA between December 2021 and April 2023. We used both the 2017 ACC/AHA and 2018 ESC/ESH thresholds to define controlled and uncontrolled arterial hypertension. We considered eleven cardiometabolic risk factors, which include overweight, obesity, central obesity, insulin resistance, diabetes, hypercholesterolemia, hypertriglyceridemia, low HDL-C, high LDL-C, low-eGFR, and high cardiovascular disease (CVD) risk.

**RESULTS:**

In a sample of 5,590 participants (female: 61%, *n* = 3,393; median age: 64 [IQR: 56–72] years), the prevalence of uncontrolled hypertension varied significantly, depending on the definition (2017 ACC/AHA: 59.9%, 95% CI: 58.6–61.2 and 2018 ESC/ESH: 20.1%, 95% CI: 19.0–21.2). In the sample, 40.43% exhibited at least 5–6 risk factors, and 32.4% had 3–4 risk factors, chiefly abdominal obesity (83.4%, 95% CI: 82.4–84.4), high LDL-C (59.6%, 95% CI: 58.3–60.9), high CVD risk (57.9%, 95% CI: 56.6–59.2), high triglycerides (56.2%, 95% CI: 54.9–57.5), and low HDL-C (42.2%, 95% CI: 40.9–43.5).

**CONCLUSIONS:**

There is a high prevalence of uncontrolled hypertension interlinked with a high burden of cardiometabolic comorbidities in Mexican adults living with arterial hypertension, underscoring the urgent need for targeted interventions and better healthcare policies to reduce the burden of the disease in our country.

## Graphical Abstract

**Graphical Abstract:**
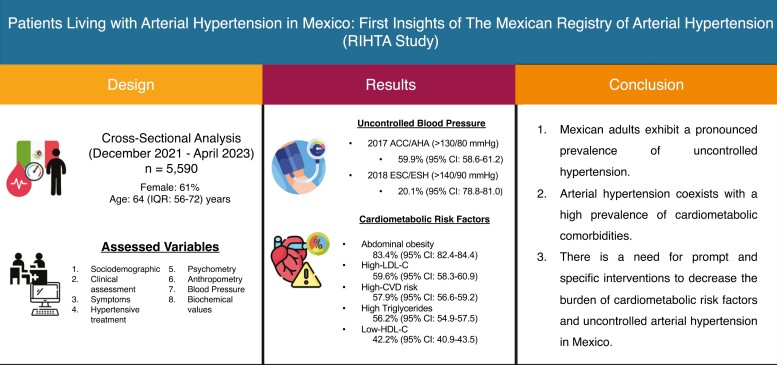

Arterial hypertension is a significant public health concern worldwide, affecting over 1.3 billion individuals and leading to more than 10 million premature deaths annually.^1,2^ As a primary contributor to atherosclerotic cardiovascular disease (ASCVD), arterial hypertension substantially affects both individual and public healthcare.^3^ Furthermore, the global prevalence of arterial hypertension is predicted to increase, particularly in low- and middle-income countries (LMICs), including Latin America.^4,5^

In Mexico, the National Health and Nutrition Survey (ENSANUT) for 2020 revealed that more than 25 million people suffer from high blood pressure.^6^ Consequently, arterial hypertension has been positioned as one of the leading causes of death and excess mortality in recent years and particularly exacerbated during the coronavirus disease 2019 (COVID-19) pandemic.^7,8^ The challenge of managing arterial hypertension in Mexico is further compounded by individual and systemic factors within the healthcare system, influencing the treatment and care of patients with this chronic condition.^9^ Despite efforts from the Mexican Group of Experts on Arterial Hypertension (Acronym in Spanish—GREHTA: Grupo de Expertos en Hipertensión Arterial) and other research groups, existing studies on hypertension’s prevalence and characteristics in Mexico remain limited and often confined to specific populations.^6,10,11^ The lack of information to deeply characterize adults with arterial hypertension poses a big challenge as these patients tend to have diverse clinical profiles and often coexist with a high burden of cardiometabolic risk factors.^12^

To address these gaps, our group established the Registry of Arterial Hypertension in Mexico (Acronym in Spanish—RIHTA: Registro de Hipertensión Arterial en México). RIHTA aspires to provide comprehensive insights into multifaceted aspects of arterial hypertension by gathering detailed information such as demographic data, clinical characteristics, laboratory results, and treatment profiles. Ultimately, RIHTA offers researchers and healthcare practitioners a vital tool to discern patterns and risk factors, evaluate treatments, and shed light on our country’s cardiometabolic risk factors related to hypertension.

Hence, in this study, we aim to give the first descriptive insights into RIHTA through two objectives: (i) assess the prevalence of controlled and uncontrolled blood pressure, and (ii) estimate the prevalence of cardiometabolic risk factors among adult patients with arterial hypertension registered between December 2021 and April 2023.

## METHODS

### Study population

**Figure 1.**
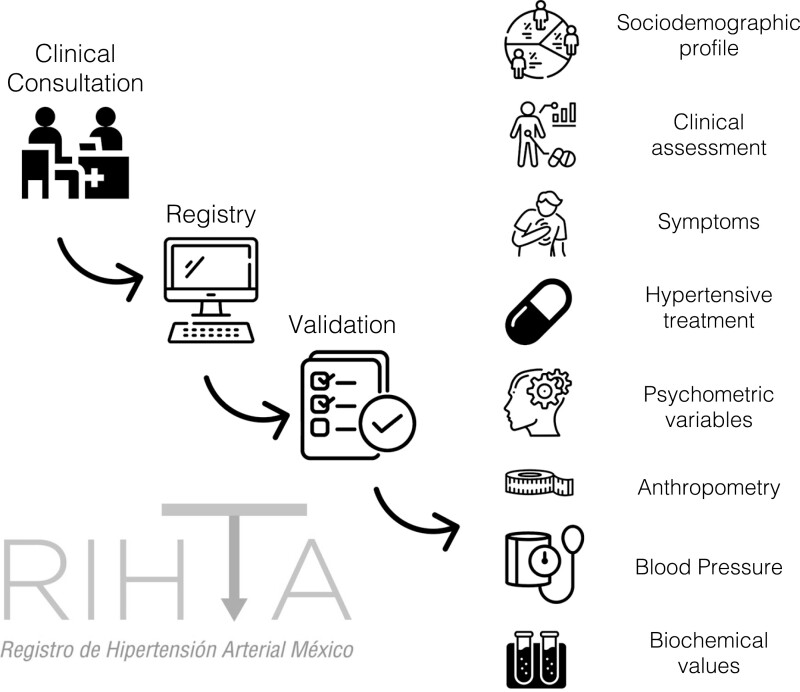
Process of information collection performed in the Registry of Arterial Hypertension in Mexico (RIHTA).

RIHTA was designed as a prospective registry and implemented as an online registry using PHP 8.0, HTML5, and MySQL 8.0 technology. The data is collected through an electronic form system with an intuitive design, requiring clarification, and validation of entered data to ensure the accuracy of the information obtained from each patient. Registered attending physicians or assistants complete data entry with online access during each office visit to supplement the medical record, including follow-up information. All information is then anonymized, and each patient receives a unique identification number. A visual diagram related to the inclusion of our patients is presented in Figure 1. For this first report, the baseline data were analyzed as a cross-sectional study from patients registered in RIHTA between December 2021 and April 2023. We included adult participants who were diagnosed with arterial hypertension as per the guidelines proposed by the World Hypertension League Expert Committee (WHLEC) and the “Norma Oficial Mexicana NOM-030-SSA2-1999".^13^ For this study, arterial hypertension was defined as meeting one of the two following criteria: (i) Systolic and/or diastolic blood pressure readings ≥140 and 90 mm Hg or (ii) having a previous clinical diagnosis of arterial hypertension and currently taking any antihypertensive treatment. We excluded patients with incomplete information to confirm the diagnosis of arterial hypertension, participants who had their residence out of Mexico, and participants who wished to withdraw their information from the registry. The medical ethics committee of the Mexican Institute of Social Security approved the protocol. All patients provided verbal informed consent before being registered in RIHTA. The study adhered to the principles of the Declaration of Helsinki and the STROBE guidelines for cross-sectional studies (Supplementary Table 1). Confidentiality of patient data was maintained in accordance with Mexican law.

### Assessed variables

RIHTA evaluated a range of variables encompassing sociodemographic profile, clinical assessment, symptoms related to arterial hypertension, hypertensive treatment, psychometric variables, anthropometry, blood pressure measurements, and biochemical values. The assessed variables included:

- *Sociodemographic profile*: Age (categorized as <45, 45–65, and ≥65 years), sex, state of residency grouped by regions, years of educational attainments (categorized as none-education, elementary, high school, college, or higher), ethnic group, social security coverage, and type of social security affiliation were considered as socioeconomic variables.
- *Clinical assessment*: This encompassed self-reported data on diabetes, previous CVD, stroke, chronic kidney disease (CKD), history of SARS-CoV-2 infection, and vaccination status up to recruitment time. Information on hypertensive disorders during pregnancy was collected for women, including self-reported preeclampsia, eclampsia, or high blood pressure in any previous pregnancy. Exercise duration, smoking status, alcohol consumption, and physician-recommended dietary changes were also asked.
- *Symptoms related to arterial hypertension:* The collected information was self-reported symptoms such as dyspnea, headache, dizziness, snoring, angina, palpitations, edema, syncope, and/or drowsiness during the last clinical visit.
- *Hypertensive treatment:* Details about the specific type of antihypertensive, regimen of treatment (e.g., monotherapy, double therapy, triple therapy, or more than four antihypertensives), and type of treatment (using only antihypertensives, only non-antihypertensive agents, or a combination) were recorded. Statin, fibrate, ezetimibe, and aspirin were also asked as additional pharmacological treatments.
- *Psychometric variables:* Participants were asked if they recognized that they lived with hypertension, the last clinical recording of blood pressure, whether they took blood pressure readings at home, where they learned to take blood pressure measurements, and if they had experienced symptoms of anxiety or sadness over the past month.
- *Anthropometry:* This included measurements taken in clinical consultation for weight, height, waist circumference in centimeters (cm), body mass index (BMI) in kg/m^2^, and heart rate (HR) in beats per minute (BPM).
- *Blood pressure* measurements: During the clinical consultation, blood pressure measurements were taken using a standardized protocol given to all physicians. This protocol consisted of taking three separate readings after the patient had rested for 5 minutes in the quietest environment possible. Patients were verbally asked to avoid drinking caffeinated beverages and smoking for 24 hours prior to their consultation. For this analysis, the final two readings were averaged. We provided a calibrated digital sphygmomanometer (OMRON Healthcare EMC Model HEM-9200T) to all clinicians in RIHTA for the BP measurements.
- *Biochemical values:* Values of hemoglobin, serum creatinine, glucose, triglycerides, total cholesterol, low-density cholesterol (LDL-C), high-density cholesterol (HDL-C), and glycated hemoglobin (HbA1c) were recorded from the 6 months preceding the last clinical visit.

### Clinical surrogates

The metabolic score for insulin resistance (METS-IR) was included as a clinical surrogate developed for the Mexican population to measure insulin resistance.^14^ The equation for METS-IR is expressed as follows: $Ln\left(\left(2*G_{0}\right)+TG_{0}\right)*BMI)/\left(Ln\left(HDL\text{-}c\right)\right)\;$; [where G_0_: fasting glucose (mg/dl), TG_0_: fasting triglycerides (mg/dl), BMI: body mass index, HDL-c: high-density lipoprotein cholesterol (mg/dl)]. The metabolic score for visceral fat estimation (METS-VF) was included as a clinical estimator of visceral adipose tissue (VAT), with the exponential transformation of METS-VF reflecting an estimation of VAT in grams.^15^ The equation for METS-VF is expressed as follows: $METS\text{-}VF=4.466+0.011\left[(Ln{\left(METS\text{-}IR\right)}^{3}\right]+3.239[Ln(waist\;circumference/$${height)}^{3}]+0.319\left(male\;sex\right)+0.594\left[Ln\left(age\right)]\right)$. The estimated glomerular filtration rate (eGFR) was calculated using the 2021 CKD-EPI equation proposed by Inker *et al*. based on serum creatinine estimations without race and applied to our sample using the “nephro” R package.^16,17^ CKD categories were classified according to the Kidney Disease Improving Global Outcomes (KDIGO) international guidelines as Stage 1 (≥90 ml/min/1.73 m²), Stage 2 (60–89 ml/min/1.73 m²), Stage 3 (30–59 ml/min/1.73 m²), Stage 4 (15–29 ml/min/1.73 m²), and Stage 5 or dialysis (<15 ml/min/1.73 m²).^18^ We estimated CVD risk for our sample using the Mexican office-based equation proposed by the Globorisk Consortium and estimated in our sample using the “globorisk” R package.^19,20^

### Blood pressure goals definitions

The first objective of this study was to estimate the prevalence of controlled and uncontrolled arterial hypertension within RIHTA. To achieve this, we used the 2017 American College of Cardiology/American Heart Association (ACC/AHA) and 2018 European Society of Cardiology/European Society of Hypertension Blood Pressure/Hypertension (ESC/ESH) Guidelines. According to the 2017 ACC/AHA, controlled arterial hypertension is defined by an SBP/DBP reading of less than 130/80 mm Hg. In contrast, the 2018 ESC/ESH Guidelines use a threshold of less than 140/90 mm Hg to characterize controlled hypertension.^21^

### Cardiometabolic risk factors definitions

The second objective of this study was centered on estimating the prevalence of 11 cardiometabolic risk factors related to the onset of CVD: (i) Overweight, defined as a BMI between 25 and 29.9 kg/m²; (ii) Obesity, defined as a BMI ≥30 kg/m^222^; (iii) Central obesity, identified as a waist circumference ≥90 cm in men and ≥80 cm in women using the International Diabetes Federation (IDF) criteria for the Mexican population^23^; (iv) Insulin Resistance, defined as a METS-IR index ≥51.13 based on the previously validated equation for Mexican population^14^; (v) Diabetes, determined by the American Diabetes Association criteria, including a previous diagnosis, fasting glucose ≥126 mg/dl, or glycated hemoglobin ≥6.5%^24^; (vi) Hypercholesterolemia, identified as total cholesterol ≥200 mg/dl according to the “2019 American College of Cardiology/American Heart Association Guideline on the Primary Prevention of Cardiovascular Disease”^25^; (vii) Hypertriglyceridemia, with TG levels ≥150 mg/dl; (viii) Low HDL-C, defined as <40 mg/dl in males or <50 mg/dl in females; (ix) High LDL-C, characterized by an LDL-C level ≥100 mg/dl^26^; (x) Low-eGFR, defined as an eGFR <60 ml/min/1.73 m^227^; and (xi) high CVD risk, defined using by a CKD-risk estimated by Globorisk equation ≥10%.^19^

### Statistical analyses

The presentation of continuous data depended on its distribution. The variables with a normal distribution were expressed as either means (standard deviation) or medians (interquartile range) if not, as determined by the Anderson–Darling normality test. Categorical variables were presented as frequency and absolute proportions. Statistical analyses were performed in R Studio (Version 4.1.2).^28^

### Missing values assessment

We assumed that the data were missing completely at random for handling missing biochemical values. We used the “*mice*” R Package (Version 3.14.0) to generate five imputed datasets, carrying out a maximum of five iterations and combining them according to Rubin’s rules.^29^ Detailed results of imputed variables can be found in the Supplementary Figure 1.

### Prevalence estimations

To estimate the prevalence of uncontrolled and controlled arterial hypertension and the cardiometabolic risk factors, along with a 95% confidence interval estimation, we used the Clopper-Pearson method from the “*epiR*” package (Version 2.0.3).^30^ The Clopper-Pearson method was selected because it has been classified as a conservative and a better approach to estimating confidence intervals in binomial distributions regardless of the sample size.^31,32^ To visualize the overall prevalence of controlled and uncontrolled arterial hypertension and cardiometabolic risk factors, use the “*ggplot2*” R Package (Version 3.4.2).^33^ As a sensitivity analysis, we performed the evaluation of the prevalence of cardiometabolic risk factors using our dataset without multiple imputations. Finally, we stratified the prevalence of uncontrolled arterial hypertension by sex, age categories, and educational attainments.

## RESULTS

Our study comprised information from 5,590 participants with arterial hypertension captured in RIHTA. Table 1 presents the complete characteristics of the study population. Briefly, the sample had a female predominance (61%, *n* = 3,393) from an adult population with a median age of 64 years (IQR: 56–72), mainly from the central region (95.8%, *n* = 5,357), with high-school education (43%, *n* = 2,430) and self-identified ethnicity as mestizo (97%, *n* = 5,411). The complete list of participants by state and region is presented in Supplementary Table 2. The 98% (*n* = 5,490) of our sample were affiliated with social security, mainly in the Mexican Institute of Social Security. Regarding the clinical profile, we observed that the most frequent concomitant comorbidity was diabetes (39%, *n* = 2,191, median HbA1c: 7.5%, IQR: 6.8–9.3), current smoking (16%, *n* = 917), and previous CVD (6.3%, *n* = 353); 1.95% (*n* = 66) of the women reported any hypertensive disorder during pregnancy. The most frequent symptoms related to arterial hypertension were headache (30%, *n* = 1,651), dizziness (20%, *n* = 1,091), and edema (14%, *n* = 773). Regarding the psychometric evaluation, most of our sample recognize and accept to live with arterial hypertension (94%, *n* = 5,236), had their last BP measurement in the last year (93%, *n* = 5,216), take their BP at home (65%, *n* = 3,647). The anthropometry of our sample comprised a median BMI of 28.7 (IQR: 25.9–32.1) kg/m^2^, a median waist circumference of 95 (IQR: 87–102) cm, a median SBP of 130 (IQR: 121–139) mm Hg and median DBP of 80 (IQR: 75–88) mm Hg. Regarding the biochemical profile, we recorded a median glucose of the total sample of 103 mg/dl (IQR: 92–128), a median HbA1c of 7.10% (IQR: 6.20–8.55), a median total cholesterol of 184 (IQR: 158–210) mg/dl, and a median of triglycerides of 158 (IQR: 121–218) mg/dl. For the clinical surrogates, we recorded a median VAT of 1,315 (IQR: 941–1,687) gr, a median eGFR of 86 (IQR: 68–99) ml/min/1.73 m², and a median CVD risk of 12% (IQR: 7–19).

**Table 1.** Descriptive characteristics of the Mexican Registry of Arterial Hypertension (RIHTA)

Characteristics	*n* = 5,590
Sociodemographic profile	
Sex, (%)	
Female	3,393 (61%)
Male	2,197 (39%)
Region, (%)	
North	117 (2.1%)
Central	5357 (95.8%)
West	22 (0.4%)
South	35 (0.6%)
Unknown	59 (1.1%)
Age, (years) [median, IQR]	64 (56, 72)
Groups of age, (%)	
<45	419 (7.5%)
45–65	2,429 (43%)
≥65	2,742 (49%)
Education, (years)	
None-education	206 (3.7%)
Elementary	2,081 (37%)
High school	2,430 (43%)
College or higher	873 (16%)
Ethnic origin, (%)	
Mestizo	5,411 (97%)
Other	179 (3.2%)
Has a social security, (%)	5,490 (98%)
Mexican Institute of Social Security, (%)	5,423 (97.0%)
Other type of social security, (%)	167 (3.0%)
Clinical assessment	
Diabetes, (%)	2,191 (39%)
Previous CVD, (%)	353 (6.3%)
Stroke, (%)	79 (1.4%)
CKD, (%)	199 (3.6%)
Previous SARS-CoV-2 infection, (%)	1,659 (30%)
COVID-19 vaccine scheme at 31 October 2022, (%)	
No	206 (3.7%)
Incomplete	487 (8.7%)
Complete	4,888 (87%)
Unknown	9 (0.6%)
Current smoking, (%)	917 (16%)
Alcoholism, (%)	17 (0.3%)
Time of exercise, (%)	
No-exercise	3,043 (54%)
<150 minutes	2,260 (40%)
≥150 minutes	281 (5.0%)
Caloric restriction, (%)	1,546 (28%)
Protein restriction, (%)	679 (12%)
Saline restriction, (%)	2,727 (49%)
Hypertensive disorder in pregnancy, (%)	66 (1.95%)
Symptoms	
Dyspnea, (%)	648 (12%)
Headache, (%)	1,651 (30%)
Dizziness, (%)	1,091 (20%)
Snoring, (%)	721 (13%)
Angina, (%)	232 (4.2%)
Palpitations, (%)	596 (11%)
Edema, (%)	773 (14%)
Syncope, (%)	88 (1.6%)
Drowsiness, (%)	509 (9.1%)
Psychometric variables	
Recognize and accept to live with arterial hypertension, (%)	5,236 (94%)
Last BP measurement, (%)	
Never	305 (5.5%)
<1 year	5,216 (93%)
>1 year	67 (1.2%)
BP is taken at home, (%)	3,647 (65%)
Where did you learn to take BP, (%)	
Learn alone	792 (14.1%)
A relative/friend taught him	1,698 (30.4%)
Taught by medic	1,085 (19.4%)
Unknown	2,015 (36%)
Anxiety last month, (%)	
No	2,667 (47.7%)
Little	1,767 (31.6%)
More-or-less	772 (13.8%)
Very	286 (5.1%)
Very-much	85 (1.52%)
Unknown	13 (0.23%)
Sadness last month, (%)	
No	2,675 (47.8%)
Little	1,783 (31.8%)
More-or-less	760 (13.6%)
Very	258 (4.6%)
Very-much	103 (1.8%)
Unknown	11 (0.19%)
Anthropometry	
BMI, (kg/m^2^) [median, IQR]	28.8 (25.9, 32.1)
Waist circumference, (cm) [median, IQR]	95 (87, 102)
SBP, (mm Hg) [median, IQR]	130 (121, 139)
DBP, (mm Hg) [median, IQR]	80 (75, 88)
HR, (bpm) [median, IQR]	75 (70, 82)
Biochemical values	
Hemoglobin, (mg/dl) [median, IQR]	14.90 (13.80, 16.01)
Serum creatinine, (mg/dl) [median, IQR]	0.84 (0.70, 1.02)
Glucose, (mg/dl) [median, IQR]	103 (92, 128)
HbA1c, (mg/dl) [median, IQR]	7.10 (6.20, 8.55)
Unknown	5,314
Total cholesterol, (mg/dl) [median, IQR]	184 (158, 210)
LDL-C, (mg/dl) [median, IQR]	110 (81, 134)
HDL-C, (mg/dl) [median, IQR]	47 (38, 58)
Triglycerides, (mg/dl) [median, IQR]	158 (121, 218)
Clinical surrogates	
METS-IR, [median, IQR]	44.64 (38.69–51.64)
Visceral adiposity [METS-VF], (%) [median, IQR]	1,315 (941, 1,687)
eGFR, (ml/min/1.73 m²) [median, IQR]	86 (68, 99)
CKD stages, (%)	
Stage 1	2,422 (43%)
Stage 2	2,202 (39%)
Stage 3	720 (13%)
Stage 4	121 (2.2%)
Stage 5	125 (2.2%)
CVD risk by Globorisk, (%)	12 (7, 19)

Abbreviations: BMI, Body Mass Index; BP, blood pressure; CKD, chronic kidney disease; CVD, cardiovascular disease; DBP, diastolic blood pressure; eGFR, estimated glomerular filtration rate; SBP, systolic blood pressure; LDL-C, low-density cholesterol.

### Treatment profiles in the studied sample

**Figure 2.**
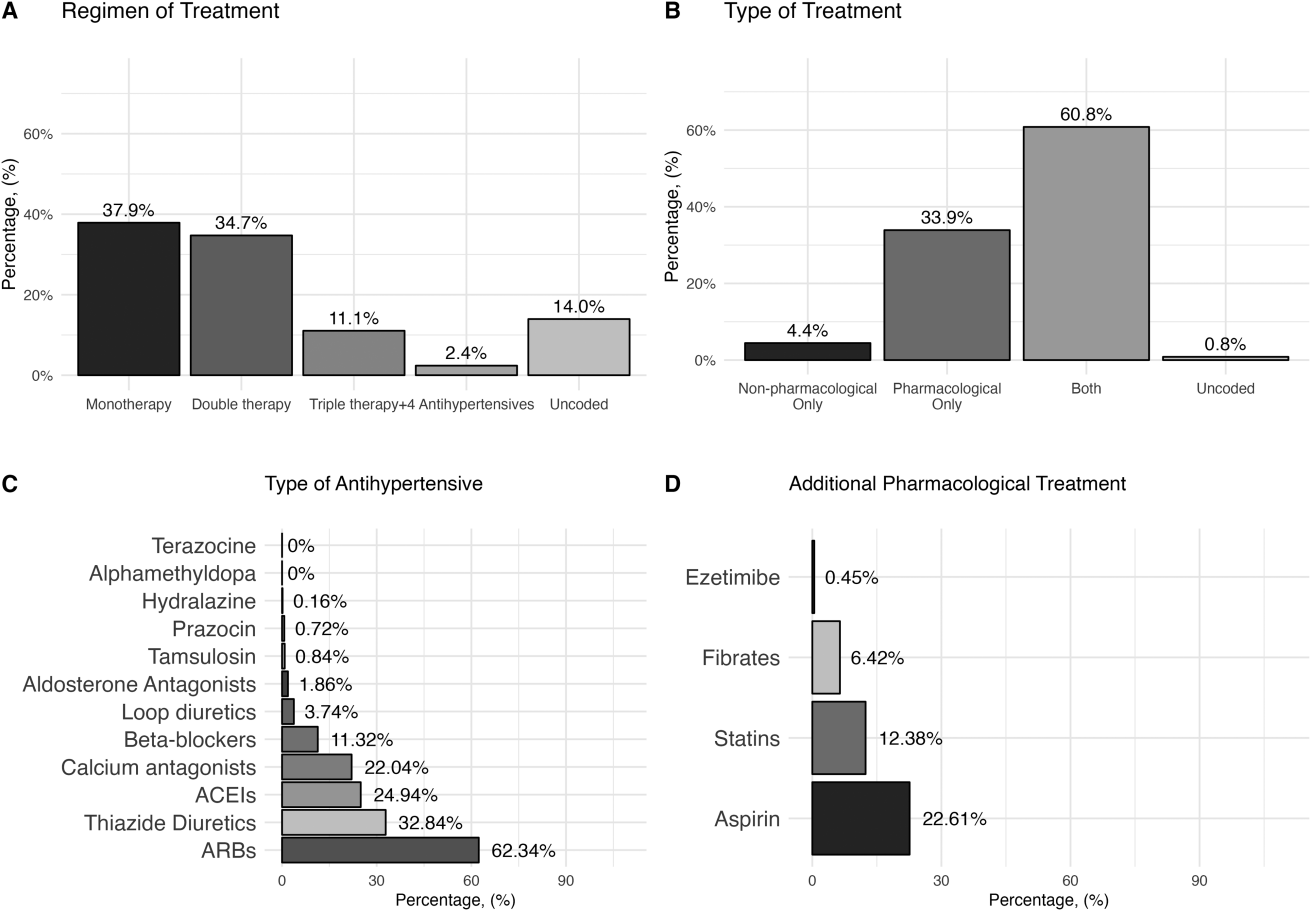
Pharmacological assessment in the Registry of Arterial Hypertension in Mexico (RIHTA). (**a**) Regimen of treatment. (**b**) Type of treatment. (**c**) Family of antihypertensive. (**d**) Additional concomitant treatment. ACEIs, Angiotensin-Converting Enzyme Inhibitors; ARBs, Angiotensin receptor blockers.

Regarding the treatment regimen, most of the participants were under monotherapy (37.9%, *n* = 2,118), double therapy (34.7%, *n* = 1,941), triple therapy (11.1%, *n* = 618), and with ≥4 antihypertensives (2.4%, *n* = 913). Almost all of the sample was in combination treatment of both pharmacological and non-pharmacological therapies (60.8%, *n* = 3,400) or only being treated with pharmacological therapies (33.9%, *n* = 1,895). Only 4.4% self-reported that they were prescribed with only non-pharmacological therapies. The types of antihypertensive drugs used were angiotensin receptor blockers (62.3%, *n* = 3,485), thiazides (32.8%, *n* = 1,836), angiotensin-converting-enzyme inhibitors (24.9%, *n* = 1,394), and calcium-channel blockers (22.04%, *n* = 1,232). Supplementary Table 3 provides a complete description of all pharmacological agents. Regarding concomitant treatment, 22.6% (*n* = 1,264) were on aspirin, 12.4% (*n* = 692) on any statin, and 6.4% (*n* = 359) with any fibrate (Figure 2).

### Evaluation of controlled and uncontrolled blood pressure

**Figure 3.**
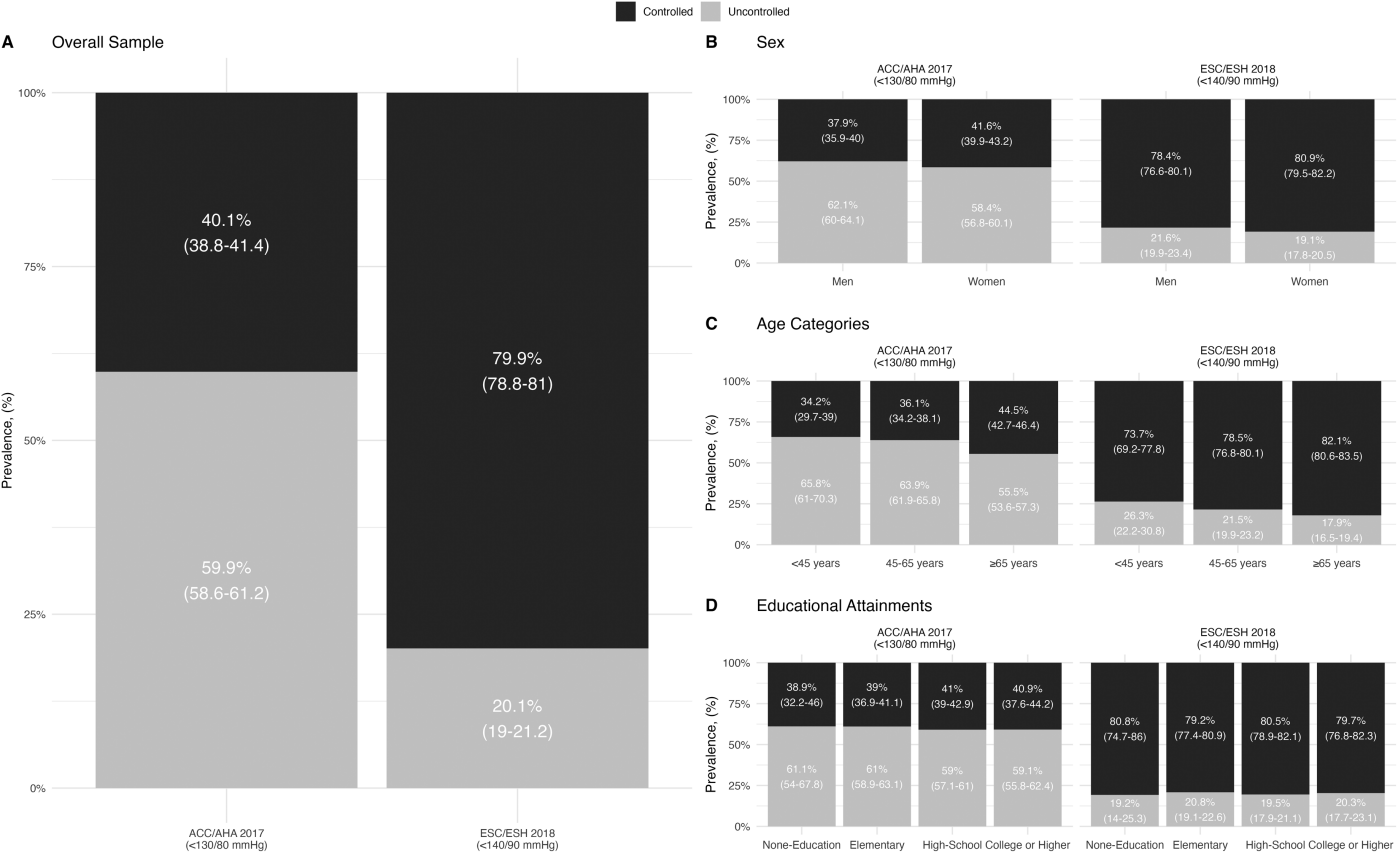
Blood pressure goals defined by the 2017 ACC/AHA and 2018 ESC/ESH hypertension guidelines in the Registry of Arterial Hypertension in Mexico. (**a**) Overall sample. (**b**) Stratification by sex. (**c**) Stratification by age categories. (**d**) Stratification by educational attainments. ACC/AHA, American College of Cardiology/American Heart Association (ACC/AHA); ESC/ESC, European Society of Cardiology/European Society of Hypertension Blood Pressure/Hypertension.

According to the 2017 ACC/AHA definition, the prevalence of uncontrolled hypertension was 59.9% (95% CI: 58.6–61.2), while controlled hypertension stood at 40.1% (95% CI: 38.8–41.4). Conversely, utilizing the 2018 ESC/ESH definition, the prevalence of uncontrolled hypertension shifted to 20.1% (95% CI: 19.0–21.2) and 79.9% (95% CI: 78.8–81.0) for controlled hypertension. Stratification revealed that men had a higher prevalence of uncontrolled hypertension compared to women. Conversely, adults aged 65 years or older showed lower rates of uncontrolled hypertension compared to younger individuals. When analyzing hypertension control in relation to educational attainments, we found no significant differences (Figure 3).

### Prevalence of cardiometabolic risk factors

**Figure 4.**
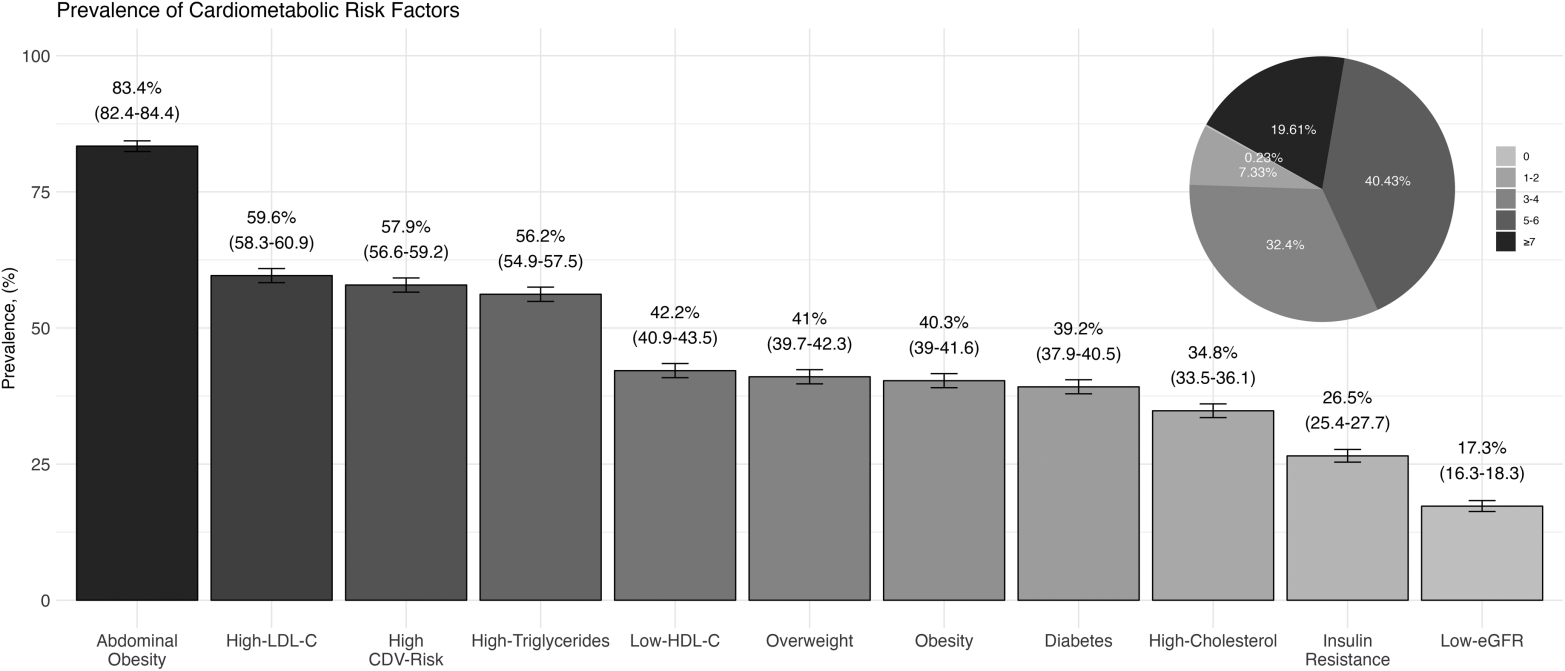
Prevalence of specific and cumulative number of cardiometabolic risk factors in the Registry of Arterial Hypertension in Mexico (RIHTA). CVD, cardiovascular disease; eGFR, estimated glomerular filtration rate; LDL-C, low-density cholesterol.

We found a high prevalence of cardiometabolic risk factors. Specifically, 40.43% of the sample exhibited at least 5–6 risk factors, and 32.4% had 3–4 risk factors. The risk factors with the highest prevalence were abdominal obesity at 83.4% (95% CI: 82.4–84.4), high LDL-C at 59.6% (95% CI: 58.3–60.9), high CVD risk at 57.9% (95% CI: 56.6–59.2), high triglycerides at 56.2% (95% CI: 54.9–57.5), and low HDL-C at 42.2% (95% CI: 40.9–43.5). Notably, 86.9% (95% CI: 86.1–87.8) had either low HDL-C, high triglycerides, or high LDL-C. Other prominent risk factors included overweight at 41.0% (95% CI: 39.7–42.3), obesity at 40.3% (95% CI: 39.0–41.6), diabetes at 39.2% (95% CI: 37.9–40.5), high cholesterol at 34.8% (95% CI: 33.5–36.1), insulin resistance at 26.5% (95% CI: 25.4–27.7), and low-eGFR at 17.3% (95% CI: 16.3–18.3) (Figure 4). The prevalence of cardiometabolic risk factors was similar in the dataset without multiple imputations (Supplementary Figure 2). Furthermore, our stratification by hypertensive management revealed that participants with uncontrolled hypertension had a significantly increased prevalence of these cardiometabolic conditions (Supplementary Figure 3).

## DISCUSSION

In this initial report from the RIHTA, we analyzed the information of 5,590 participants with arterial hypertension with the aim of giving the first comprehensive insights into the characteristics, prevalence of uncontrolled and controlled hypertension, and cardiometabolic risk factors within this registry. We found that Mexican patients living with arterial hypertension exhibited distinct sociodemographic, clinical, pharmacological, biochemical, and cardiometabolic profiles. These attributes coexist with a high prevalence of uncontrolled hypertension and a high burden of cardiometabolic comorbidities.

### Characteristics of adults living with hypertension in Mexico

In our study of adults living with hypertension in Mexico, we examined a population with distinct socio-economic characteristics, predominantly middle-urban class workers with low educational attainments. Furthermore, it is a population characterized by a female predominance, mainly made up of adults over 60 years of age, in which only 7.5% are under 45 years of age, which corresponds to the sociodemographic profile described for the LATAM population. Additionally, nearly all participants were affiliated with the Mexican Social Security Institute.^34^ According to the ENSANUT 2022 survey, around 51.2% reported having social security coverage.^35^ The Mexican Institute of Social Security covers up to 80% of its affiliated population, equating to approximately 62 million people. This overall indicates health coverage in our population, although specific fees may apply depending on the state or region and the inclusion of participants.^34^ Regarding the treatment profiles, we found a diversity in the overall scheme and profiles; over half of the patients in RIHTA combine pharmacological and non-pharmacological therapies, a small portion relies solely on non-pharmacological interventions, while roughly a third utilizes only pharmacological therapies. Furthermore, the use of monotherapy in 37.9% of participants, double therapy in 34.7%, triple therapy in 11.1%, and four or more antihypertensives in 2.4% could reflect a stratified approach to treatment complexity. Our findings reveal a significant portion of the population on monotherapy with uncontrolled BP, highlighting gaps in adherence to updated therapeutic guidelines and access to combination medications. This has been described in Mexico, where hypertension management does not rely solely on one single treatment as there is a substantial combination of double antihypertensive medication, as previously reported.^11^ Commonly prescribed medications include angiotensin receptor blockers in most cases, followed by thiazides, angiotensin-converting-enzyme inhibitors, and calcium-channel blockers, which overall results in a diversity of treatment modalities. This is further exacerbated by the inadequate prescription of statins, ezetimibe, and fibrates, given the high prevalence of diabetes and dyslipidemias reported in our sample. Despite clear institutional protocols, the widespread use of daily aspirin reveals underlying systemic challenges in managing these complex and interrelated risk factors. Future efforts by our study group will be centered on implementing educational strategies targeted at healthcare providers and patients in primary care settings, aiming to enhance BP management and improve BP goals.

### Prevalence of uncontrolled arterial hypertension in Mexico

Notably, 59.9% exhibited uncontrolled hypertension according to the 2017 ACC/AHA threshold, and 20.1% based on the 2018 ESC/ESH guidelines. To understand the broader context of arterial hypertension in Mexico, we compared our findings with local studies, which typically focus on younger populations and various health systems, primarily emphasizing the prevalence of uncontrolled hypertension, although they lack detailed information on comorbidities and specific treatments (Table 2). Interestingly, control rates in our RIHTA study are higher than the 53.9% found in the global 2021 May-Measurement Month (MMM) report, where our group played a significant role.^41^ Our results are consistent with reports made in the USA, where control rates were around 69% from 2009 to 2020.^42^ Another study by Kaiser Permanente et al. also reported an improvement in hypertension control from 44% to 90% over 13 years (2000–2013).^43^ This alignment with the U.S. data is further substantiated using a threshold of 130/80 mm Hg where 40.1% of our sample had controlled rates.^43^ Intriguingly, these control levels exceed those reported in various official surveys in Mexico but align with findings from the MMM study, underscoring the nuanced landscape of hypertension control in Mexico.^10,40,41^

**Table 2.** Proportion of people in BP goals thought representative surveys according to the 2017 ACC/AHA (<140/90 mm Hg) threshold definition in Mexico

	ENSA 2000^36^ (%)	ENSANUT 2012^37^ (%)	ENSANUT M.C. 2016^38^ (%)	ENSANUT 2020^6^ (%)	ENSANUT-2022 ^39^ (%)	MMM-18^40^ (%)	MMM-19^10^ (%)	RIHTA-2023 (%)
Proportion of people in BP goals (<140/90 mm Hg)	14.6	52.0	58.7	54.9	59.4	66.5	66.8	79.9

Abbreviations: ENSA, National Health Survey; ENSANUT, National Health and Nutrition Survey; MMM, May-measurement month survey; RIHTA, Registry of Arterial Hypertension in Mexico.

### Clinical impact of cardiometabolic risk factors in Mexico

RIHTA findings reinforce the complex nature of hypertension and the clinical impact of cardiometabolic risk factors in Mexico. We estimated a prevalence of combined overweight and obesity of 83% in our sample, which is considerably higher than the 75.2% reported by the National Health and Nutrition Survey 2022 for the general population.^44^ This should be interpreted as these patients tend to have an increased weight, which could exacerbate hypertension management and pose a risk for adverse complications. The high prevalence of both overweight and obesity has been recognized to be a health problem for the general population and for patients living with chronic health diseases, which drives the implementation of targeted interventions to decrease the burden of both diseases in our country.^45^ The apparent disconnect between dietary control and therapeutic lifestyle modifications is another intriguing discovery, with only 28% stating caloric intake restriction despite 61% receiving relevant advice. This finding reinforces the notion that there is a low emphasis on non-pharmacological approaches to manage hypertension in Mexico.^46^ In Mexico, information on lipid levels is often scarce and dispersed, yet our study revealed inadequate lipid levels in 87% of our population, with various imbalances in LDL-C, HDL-C, and triglycerides as reported in national surveys.^47^ Furthermore, the high prevalence of diabetes in our sample, at 39%, is more than double the national figure, with alarming levels of average HbA1c and insulin resistance.^48^ Overall, the high prevalence of lipid and glucose disturbances is a problem of concern given that both conditions exacerbate the risk for CVD.^49^ Meanwhile, smoking prevalence was slightly lower in our sample, but kidney damage was alarmingly higher, emphasizing the urgent need to address kidney health, which accounts for over 11,000 deaths annually in Mexico.^50^ A sedentary lifestyle is another significant problem, with 54% reporting no physical activity, a trend that compounds the issue of a high 10-year cardiovascular risk of 12%, as calculated by the Globorisk equation for the Mexican population.^51^ This data paints a picture of a multifactorial and high-burden cardiometabolic landscape, underscoring the critical need for integrated healthcare strategies to address these pressing public health issues in Mexico. Furthermore, the insights derived from the registry can guide public health initiatives, inform educational campaigns, and foster collaborations in research, thereby contributing to a more informed and effective approach to managing arterial hypertension and reducing the burden of this disease in our country.

### Strengths and limitations

Our study has both strengths and limitations that we acknowledge. RIHTA provides comprehensive and nuanced insights into a broad series of evaluations that led us to better characterize patients living with hypertension, the prevalence of uncontrolled and controlled blood pressure, and relevant cardiometabolic risk factors. Furthermore, by facilitating the tracking of longitudinal data, RIHTA is a tool to develop prospective studies that explore causal relationships regarding contributors of hypertensive-related outcomes. Nevertheless, we must acknowledge some limitations. First, RIHTA is allocated mostly to patients affiliated with the Mexican Institute of Social Security within states in the central region. This could lead to a selection bias and may not be representative of the entire Mexican population with arterial hypertension treated in other healthcare systems in Mexico. Furthermore, the patients in the registry who are affiliated with the Mexican Institute of Social Security may possess inherent characteristics that make them more likely to participate in our registry, leading to a healthier lifestyle and favorable clinical outcomes. To address this limitation, the GREHTA group invites all physicians in Mexico to be part of RIHTA to expand and report the situation of patients living with hypertension in all care settings. Second, the data collection relied mostly on self-reported information for certain variables, such as comorbidities, lifestyle habits, and medication adherence, which may be subject to recall and social desirability biases even though a trained physician captured the information in our system. Third, we were not able to include genetic, imaging, and metabolomic results, given that most of these measurements are not widely available in most primary care settings in our country, leading to a big area of opportunity to study.

## CONCLUSIONS

In conclusion, RIHTA provides a comprehensive insight into the characteristics of patients living with arterial hypertension in Mexico. The findings highlight a relatively high prevalence of uncontrolled hypertension interlinked with a high burden of cardiometabolic comorbidities, such as abdominal obesity, dyslipidemia, and high CVD risk, which overall results in an urgent need for targeted healthcare interventions. Overall, RIHTA is an invaluable tool to guide personalized, evidence-based care strategies, promote prevention, and foster local and international research collaborations toward improving hypertensive care management in the Mexican population.

Mexican Group of Experts on Arterial Hypertension

The Mexican Group of Experts on Arterial Hypertension is composed by: Silvia Palomo-Piñón, Neftali Eduardo Antonio-Villa, Luis Rey García-Cortés, Moises Moreno-Noguez, Luis Alcocer, Humberto Álvarez-López, Ernesto G. Cardona-Muñoz, Adolfo Chávez-Mendoza, Enrique Díaz-Díaz, Héctor Galván-Oseguera, Martín Rosas-Peralta, María de los Ángeles Dichi Romero, Pedro Luis Vargas Gutiérrez, María Eugenia Figueroa Suárez, Rubén Rios Morales, Francisco Vargas Hernández, Irma Fabiola García Padilla, Alfonso Zempoalteca Morales, Imer Guillermo Herrera Olvera, Gloria Mendoza López, Ana Laura Guerrero Morales, María Elisa López Delgado, Ana Lilia Gonzales Ramírez, Jairo Enoc Cruz Toledo, Olivia Reyes Jiménez, Diana Amaya Mora, Isaac Pérez Zamora, Flor Araceli Nava Ayala, Tabata Gabriela Anguiano Velázquez, Oscar Jiménez Jalpa, Ma. Adriana Cruz Arce and Vidal José González Coronado.

## Supplementary Material

hpae024_suppl_Supplementary_Tables_S1-S3_Figures_S1-S3

## Data Sharing

The dataset presented in this article is property of the Mexican Group of Experts on Arterial Hypertension. The data underlying this article will be shared on reasonable request to the corresponding author. Requests to access the dataset should be directed to: silvia-palomo@hotmail.com. R code to reproduce the results is accessible at: https://github.com/neftalivilla/RIHTA_GREHTA.
