# Supplementary material for: Patients Living With Arterial Hypertension in Mexico: First Insights of The Mexican Registry of Arterial Hypertension (RIHTA Study)
Source: Am J Hypertens. 2024 Mar 11;37(7):503–13. doi: 10.1093/ajh/hpae024 (PMC11176274; doi:10.1093/ajh/hpae024)
Supplement: hpae024_suppl_Supplementary_Tables_S1-S3_Figures_S1-S3 [file hpae024_suppl_supplementary_tables_s1-s3_figures_s1-s3.docx]

**Supplementary Material**

**Patients Living with Arterial Hypertension in Mexico: First Insights of The Mexican Registry of Arterial Hypertension (RIHTA Study)**

Silvia Palomo-Piñón MD PhD, Neftali Eduardo Antonio-Villa MD PhD, Luis Rey García-Cortés MD PhD, Luis Alcocer MD MsC, Humberto Álvarez López MD MsC, Ernesto G. Cardona-Muñoz MD, Adolfo Chávez Mendoza MD MsC, Enrique Díaz Díaz MD, Héctor Galván Oseguera MD and Martin Rosas Peralta MD PhD on behalf of the Mexican Group of Experts on Arterial Hypertension.

[Supplementary Figure 1 2](#_Toc156734230)

[Supplementary Figure 2 3](#_Toc156734231)

[Supplementary Figure 3 4](#_Toc156734232)

[Supplementary Table 1: 5](#_Toc156734233)

[Supplementary Table 2 7](#_Toc156734234)

[Supplementary Table 3 8](#_Toc156734235)

Supplementary Figure 1: We used the mice R Package (Version 3.14.0) to impute continuous missing values, assuming that data were missing completely at random ^1^. The imputation was done using multiple chained equations, and we created five imputed datasets. We combined the imputed datasets using Rubin's rules for a maximum of five iterations. We report the percentage of missing values for continuous variables (A), as well as density histograms (B) and summary statistics (C) for both the original and imputed variables. We found no statistically significant differences between the imputed variables and the original distribution of the missing continuous variables.

## Supplementary Figure 2

Prevalence of specific and cumulative number of cardiometabolic risk factors in the Registry of Arterial Hypertension in Mexico (RIHTA) dataset without multiple imputations.


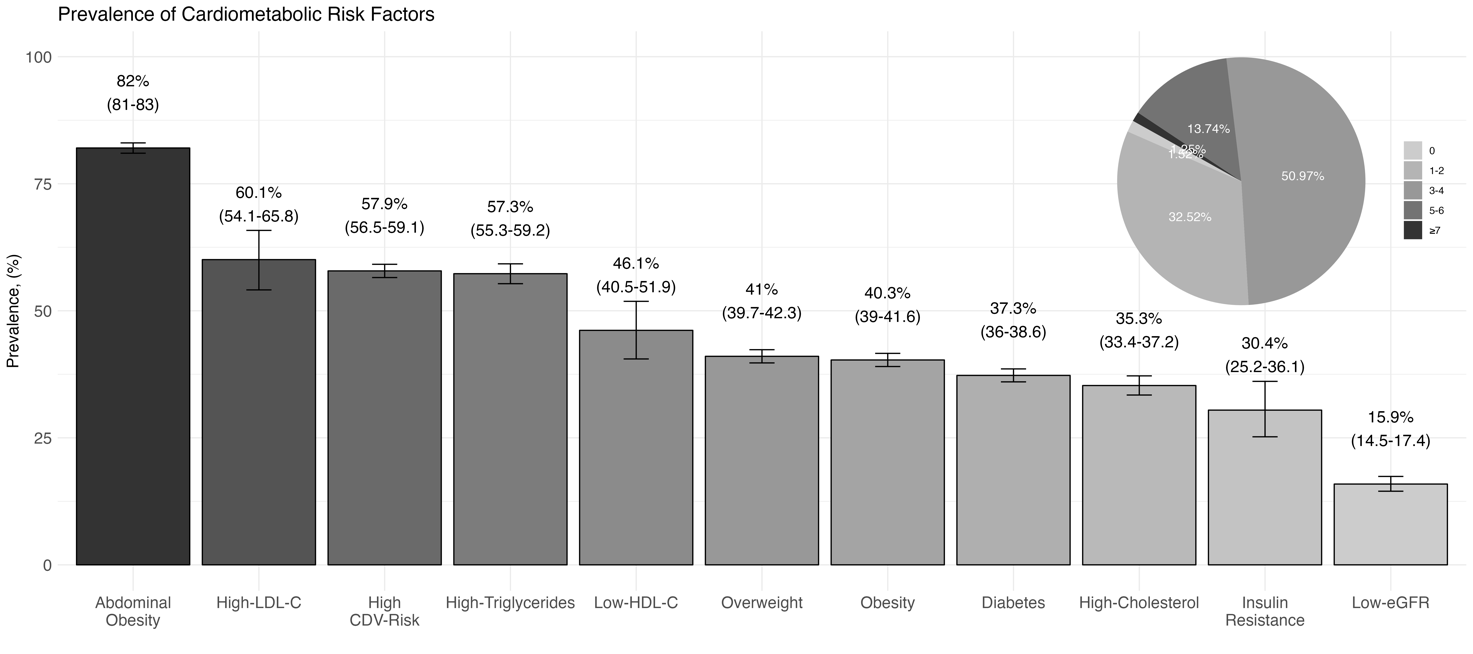


Footnote: The available number of participants with information to estimate the cardiometabolic risk factors is presented as follows: Abdominal Obesity: n= 5590; High-LDL-C: n= 283; High-CVD-Risk: n= 5570; High-Triglycerides: n= 2508; Low-HDL-C: n= 312; Overweight: n= 5580; Obesity: n= 5580; Diabetes: n= 5590; High Cholesterol: n= 2522; Insulin Resistance: n= 289; Low-eGFR: n= 2490.

Supplementary Figure 3 Prevalence of specific and cumulative number of cardiometabolic risk factors stratified by ACC/AHA 2017 and ESC/ESH 2018


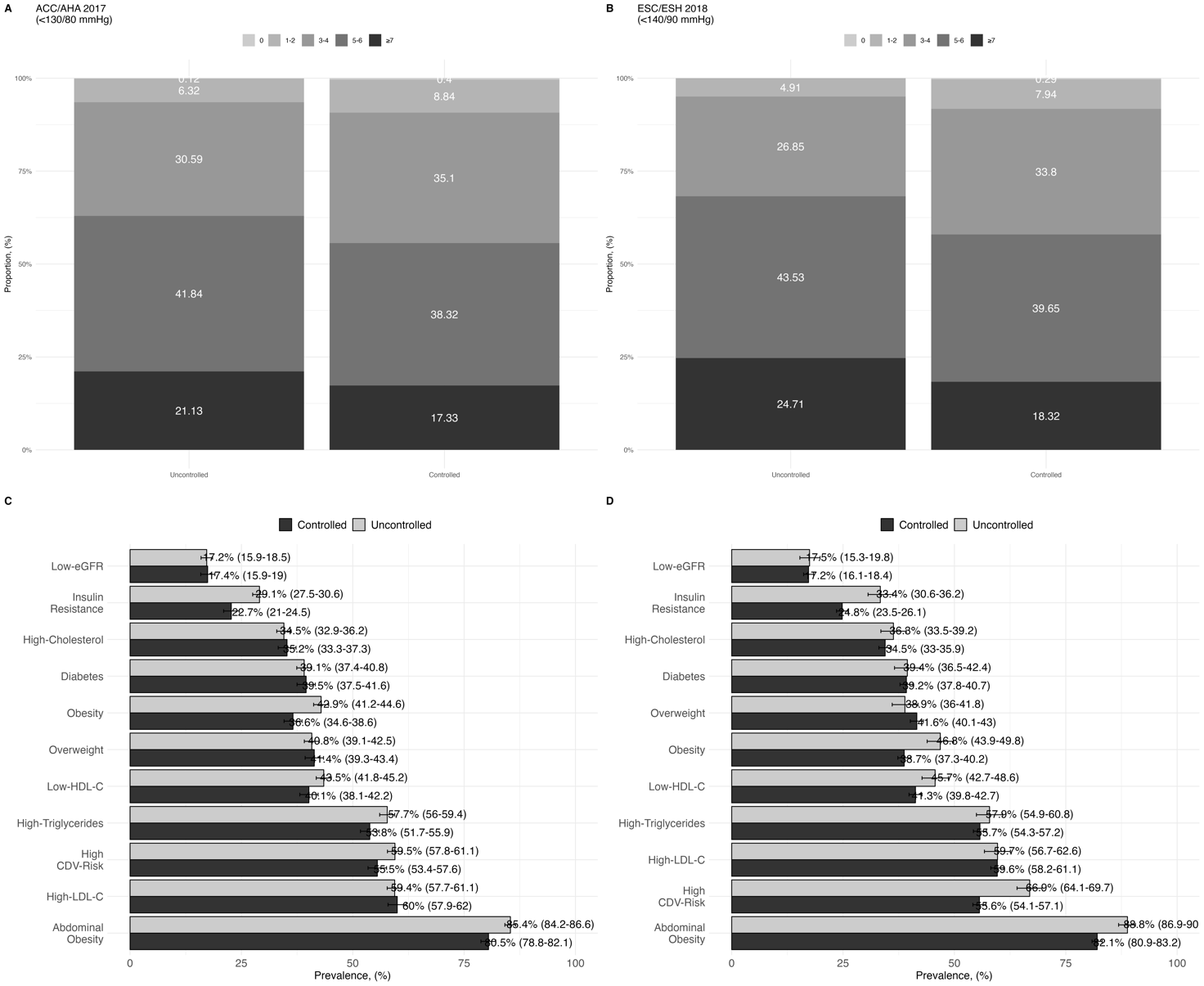


Supplementary Table 1: STROBE cross-sectional guidelines report for study. This checklist was completed on August 4^st^, 2023 using <https://www.goodreports.org/> a tool made by the EQUATOR Network in collaboration with Penelope.ai (von Elm E, Altman DG, Egger M, Pocock SJ, Gotzsche PC, Vandenbroucke JP. The Strengthening the Reporting of Observational Studies in Epidemiology (STROBE) Statement: guidelines for reporting observational studies)

|  |  | Reporting Item | Page Number |
| --- | --- | --- | --- |
| **Title and abstract** |  |  |  |
| Title | [#1a](https://www.goodreports.org/reporting-checklists/strobe-cross-sectional/info/#1a) | Indicate the study’s design with a commonly used term in the title or the abstract | 1 |
| Abstract | [#1b](https://www.goodreports.org/reporting-checklists/strobe-cross-sectional/info/#1b) | Provide in the abstract an informative and balanced summary of what was done and what was found | 4 |
| **Introduction** |  |  |  |
| Background / rationale | [#2](https://www.goodreports.org/reporting-checklists/strobe-cross-sectional/info/#2) | Explain the scientific background and rationale for the investigation being reported | 5 |
| Objectives | [#3](https://www.goodreports.org/reporting-checklists/strobe-cross-sectional/info/#3) | State specific objectives, including any prespecified hypotheses | 6 |
| **Methods** |  |  |  |
| Study design | [#4](https://www.goodreports.org/reporting-checklists/strobe-cross-sectional/info/#4) | Present key elements of study design early in the paper | 6 |
| Setting | [#5](https://www.goodreports.org/reporting-checklists/strobe-cross-sectional/info/#5) | Describe the setting, locations, and relevant dates, including periods of recruitment, exposure, follow-up, and data collection | 6-7 |
| Eligibility criteria | [#6a](https://www.goodreports.org/reporting-checklists/strobe-cross-sectional/info/#6a) | Give the eligibility criteria, and the sources and methods of selection of participants. | 6 |
|  | [#7](https://www.goodreports.org/reporting-checklists/strobe-cross-sectional/info/#7) | Clearly define all outcomes, exposures, predictors, potential confounders, and effect modifiers. Give diagnostic criteria, if applicable | 8-9 |
| Data sources / measurement | [#8](https://www.goodreports.org/reporting-checklists/strobe-cross-sectional/info/#8) | For each variable of interest give sources of data and details of methods of assessment (measurement). Describe comparability of assessment methods if there is more than one group. Give information separately for for exposed and unexposed groups if applicable. | 7 |
| Bias | [#9](https://www.goodreports.org/reporting-checklists/strobe-cross-sectional/info/#9) | Describe any efforts to address potential sources of bias | NA |
| Study size | [#10](https://www.goodreports.org/reporting-checklists/strobe-cross-sectional/info/#10) | Explain how the study size was arrived at | 9 |
| Quantitative variables | [#11](https://www.goodreports.org/reporting-checklists/strobe-cross-sectional/info/#11) | Explain how quantitative variables were handled in the analyses. If applicable, describe which groupings were chosen, and why | 7-8 |
| Statistical methods | [#12a](https://www.goodreports.org/reporting-checklists/strobe-cross-sectional/info/#12a) | Describe all statistical methods, including those used to control for confounding | 9-10 |
| Statistical methods | [#12b](https://www.goodreports.org/reporting-checklists/strobe-cross-sectional/info/#12b) | Describe any methods used to examine subgroups and interactions | 10 |
| Statistical methods | [#12c](https://www.goodreports.org/reporting-checklists/strobe-cross-sectional/info/#12c) | Explain how missing data were addressed | 10 |
| Statistical methods | [#12d](https://www.goodreports.org/reporting-checklists/strobe-cross-sectional/info/#12d) | If applicable, describe analytical methods taking account of sampling strategy | NA |
| Statistical methods | [#12e](https://www.goodreports.org/reporting-checklists/strobe-cross-sectional/info/#12e) | Describe any sensitivity analyses | NA |
| **Results** |  |  |  |
| Participants | [#13a](https://www.goodreports.org/reporting-checklists/strobe-cross-sectional/info/#13a) | Report numbers of individuals at each stage of study—eg numbers potentially eligible, examined for eligibility, confirmed eligible, included in the study, completing follow-up, and analysed. Give information separately for for exposed and unexposed groups if applicable. | 10-11 |
| Participants | [#13b](https://www.goodreports.org/reporting-checklists/strobe-cross-sectional/info/#13b) | Give reasons for non-participation at each stage | NA |
| Participants | [#13c](https://www.goodreports.org/reporting-checklists/strobe-cross-sectional/info/#13c) | Consider use of a flow diagram | NA |
| Descriptive data | [#14a](https://www.goodreports.org/reporting-checklists/strobe-cross-sectional/info/#14a) | Give characteristics of study participants (eg demographic, clinical, social) and information on exposures and potential confounders. Give information separately for exposed and unexposed groups if applicable. | 10-11 |
| Descriptive data | [#14b](https://www.goodreports.org/reporting-checklists/strobe-cross-sectional/info/#14b) | Indicate number of participants with missing data for each variable of interest | ST2 |
| Outcome data | [#15](https://www.goodreports.org/reporting-checklists/strobe-cross-sectional/info/#15) | Report numbers of outcome events or summary measures. Give information separately for exposed and unexposed groups if applicable. | 11-12 |
| Main results | [#16a](https://www.goodreports.org/reporting-checklists/strobe-cross-sectional/info/#16a) | Give unadjusted estimates and, if applicable, confounder-adjusted estimates and their precision (eg, 95% confidence interval). Make clear which confounders were adjusted for and why they were included | 11-12 |
| Main results | [#16b](https://www.goodreports.org/reporting-checklists/strobe-cross-sectional/info/#16b) | Report category boundaries when continuous variables were categorized | 10-12 |
| Main results | [#16c](https://www.goodreports.org/reporting-checklists/strobe-cross-sectional/info/#16c) | If relevant, consider translating estimates of relative risk into absolute risk for a meaningful time period | NA |
| Other analyses | [#17](https://www.goodreports.org/reporting-checklists/strobe-cross-sectional/info/#17) | Report other analyses done—e.g., analyses of subgroups and interactions, and sensitivity analyses | NA |
| **Discussion** |  |  |  |
| Key results | [#18](https://www.goodreports.org/reporting-checklists/strobe-cross-sectional/info/#18) | Summarise key results with reference to study objectives | 12-15 |
| Limitations | [#19](https://www.goodreports.org/reporting-checklists/strobe-cross-sectional/info/#19) | Discuss limitations of the study, taking into account sources of potential bias or imprecision. Discuss both direction and magnitude of any potential bias. | 15 |
| Interpretation | [#20](https://www.goodreports.org/reporting-checklists/strobe-cross-sectional/info/#20) | Give a cautious overall interpretation considering objectives, limitations, multiplicity of analyses, results from similar studies, and other relevant evidence. | 12-15 |
| Generalisability | [#21](https://www.goodreports.org/reporting-checklists/strobe-cross-sectional/info/#21) | Discuss the generalisability (external validity) of the study results | 15 |
| **Other Information** |  |  |  |
| Funding | [#22](https://www.goodreports.org/reporting-checklists/strobe-cross-sectional/info/#22) | Give the source of funding and the role of the funders for the present study and, if applicable, for the original study on which the present article is based | 17 |

Supplementary Table 2: Frequency and percentage of participants by state and region registered in the Registry of Arterial Hypertension (RIHTA).

| State | Frequency | Percentage (%) | Region |
| --- | --- | --- | --- |
| Unknown | 59 | 1.05 | Central |
| Guanajuato | 36 | 0.64 | Central |
| San Luis Potosí | 50 | 0.89 | Central |
| Morelos | 1 | 0.02 | Central |
| Tlaxcala | 4 | 0.07 | Central |
| Querétaro | 42 | 0.75 | Central |
| Puebla | 44 | 0.78 | Central |
| Hidalgo | 45 | 0.80 | Central |
| Mexico City | 114 | 2.02 | Central |
| State of Mexico | 5,020 | 89.10 | Central |
| Aguascalientes | 1 | 0.02 | Central |
| Tamaulipas | 1 | 0.02 | North |
| Jalisco | 57 | 1.01 | North |
| Coahuila | 3 | 0.05 | North |
| Durango | 5 | 0.09 | North |
| Baja California | 5 | 0.09 | North |
| Nuevo León | 6 | 0.11 | North |
| Sinaloa | 42 | 0.75 | North |
| Baja California Sur | 55 | 0.98 | North |
| Sonora | 1 | 0.02 | North |
| Quintana Roo | 1 | 0.02 | South |
| Yucatán | 6 | 0.11 | South |
| Veracruz | 7 | 0.12 | South |
| Tabasco | 9 | 0.16 | South |
| Oaxaca | 12 | 0.21 | South |
| Michoacán | 6 | 0.11 | West |
| Guerrero | 2 | 0.04 | West |

Supplementary Table 3: Description of prescription usage in the Registry of Arterial Hypertension (RIHTA) stratified by controlled and uncontrolled arterial hypertension status.

| **Characteristic** | **N = 5,590**^1^ |
| --- | --- |
| **Calcium antagonists, (%)** | 1,232 (22%) |
| **Type of calcium antagonists, (%)** |  |
| Amlodipine | 835 (68%) |
| Diltiazem | 4 (0.3%) |
| Felodipine | 4 (0.3%) |
| Nicardipine | 2 (0.2%) |
| Niferdipine | 358 (29%) |
| Verapamil | 22 (1.8%) |
| Unknown | 7 (0.6%) |
| **ACEIs, (%)** | 1,394 (25%) |
| **Type of ACEIs, (%)** |  |
| Captopril | 78 (5.6%) |
| Enalapril | 1,199 (86%) |
| Fosinorpil | 1 (<0.1%) |
| Lisinopril | 3 (0.2%) |
| Perindopril | 91 (6.5%) |
| Ramipril | 1 (<0.1%) |
| Zofenopril | 5 (0.4%) |
| Unknown | 16 (1.1%) |
| **ARBs, (%)** | 3,485 (62%) |
| **Type of ARBs, (%)** |  |
| Azilsartan | 6 (0.2%) |
| Candersartan | 8 (0.2%) |
| Eporsartan | 4 (0.1%) |
| Irbesartan | 886 (25%) |
| Losartan | 2,219 (64%) |
| Olmesartan | 7 (0.2%) |
| Telmisartan | 276 (7.9%) |
| Valsartan | 71 (2.0%) |
| Fimasartan | 4 (0.1%) |
| Unknown | 4 (0.1%) |
| **Thiazide Diuretics, (%)** | 1,836 (33%) |
| **Type of thiazide diuretics, (%)** |  |
| Clortalidona | 130 (7.1%) |
| Hydrochlorothiazide | 1,648 (90%) |
| Indapamide | 55 (3.0%) |
| Unknown | 3 (0.2%) |
| **Aldosterone Antagonists, (%)** | 104 (1.9%) |
| **Type of aldosterone antagonists, (%)** |  |
| Spironolactone | 100 (96%) |
| Unknown | 4 (3.8%) |
| **Loop diuretics, (%)** | 209 (3.7%) |
| **Type of loop diuretics, (%)** |  |
| Furosemide | 196 (94%) |
| Bumethamide | 1 (0.5%) |
| Unknown | 12 (5.7%) |
| **Beta−blockers, (%)** | 633 (11%) |
| **Type of beta−blockers, (%)** |  |
| Atenolol | 4 (0.6%) |
| Bisoprolol | 8 (1.3%) |
| Metoprolol Succinate | 77 (12%) |
| Metoprolol Tartate | 493 (78%) |
| Nebivolol | 4 (0.6%) |
| Propanolol | 45 (7.1%) |
| Carvedidol | 1 (0.2%) |
| Unknown | 1 (0.2%) |
| **Prazocin, (%)** | 40 (0.7%) |
| **Tamsulosin, (%)** | 47 (0.8%) |
| **Hydralazine, (%)** | 9 (0.2%) |
| **Aspirin, (%)** | 1,264 (23%) |
| **Statins, (%)** | 692 (12%) |
| **Type of statin, (%)** |  |
| Rosuvastatin | 22 (3.2%) |
| Atorvastatin | 447 (65%) |
| Pravastatin | 216 (31%) |
| Unknown | 7 (1.0%) |
| **Ezetimibe, (%)** | 25 (0.4%) |
| **Type of ezetimibe, (%)** |  |
| Ezetimibe | 19 (76%) |
| Ezetimibe/Simvastatin | 5 (20%) |
| Unknown | 1 (4.0%) |
| **Fibrates, (%)** | 359 (6.4%) |
| **Type of fibrates, (%)** |  |
| Fenofibrate | 7 (1.9%) |
| Bezafibrate | 253 (70%) |
| Cipofibrate | 53 (15%) |
| Unknown | 46 (13%) |
| ^1^n (%) | |

*Abbreviations*: ACEI: Angiotensin-converting-enzyme inhibitors; ARB: Angiotensin receptor blockers; CCB: Calcium channel antagonists
